# HSP90 inhibition downregulates thymidylate synthase and sensitizes colorectal cancer cell lines to the effect of 5FU-based chemotherapy

**DOI:** 10.18632/oncotarget.2484

**Published:** 2014-09-16

**Authors:** Ganji Purnachandra Nagaraju, Olatunji B. Alese, Jerome Landry, Roberto Diaz, Bassel F El-Rayes

**Affiliations:** ^1^ Department of Hematology and Medical Oncology, Atlanta, USA; ^2^ Department of Radiation Oncology Emory University, Atlanta, GA; ^3^ Department of Radiation Oncology, H. Lee Moffitt Cancer Center & Research Institute, Tampa, FL, USA

**Keywords:** Colon cancer, ganetespib, Hsp90, cell cycle progression and chemotherapy

## Abstract

Cell cycle progression and DNA synthesis are essential steps in cancer cell growth. Thymidylate synthase (TS) is a therapeutic target for 5FU. We tested the hypothesis that HSP90 transcriptional and functional inhibition can inhibit cell cycle progression, downregulate TS levels and sensitize colorectal cancer (CRC) cell lines to the effects of 5FU. Treatment with ganetespib (50nM) for 24 hours inhibited cyclin D1 and pRb at the transcriptional and translational levels and induced p21, leading to G0/G1 cell cycle arrest in both CRC cell lines (HCT-116 and HT-29). This was associated with downregulation of E2F1 and its target gene TS. In addition, ganetespib inhibited PI3K/Akt and ERK signalling pathways. Similar effects were observed with HSP90 knockdown in both cell lines. Ganetespib sensitized CRC cell lines to the effects of oxaliplatin and 5FU. Similar effects were also observed in tumors from animals treated with ganetespib, oxaliplatin and 5FU. In this study, we present *in vitro* and animal data supporting that the targeting of HSP90 decreases CRC cell survival and proliferation. Ganetespib sensitizes CRC cell lines to the effects of 5FU-based chemotherapy. Combining HSP90 inhibitors with chemotherapy is a rational approach for future drug development in CRC.

## INTRODUCTION

Colorectal cancer (CRC) is the second leading cause of mortality in the United States [1]. Approximately 30% of patients with CRC present with advanced stage disease [2]. Although a significant improvement in overall survival has been observed in patients with stage IV CRC, the five year survival rate remains below 20% which highlights the importance of developing newer and more effective systemic therapies [1]. *De novo* or acquired resistance to systemic chemotherapy regimens remains a major challenge in the management of CRC. Fluoropyrimidine (5FU)-based chemotherapy remains the treatment of choice for this group of patients [3]. 5FU is a nucleotide analogue that inhibits thymidylate synthase (TS), a key enzyme in the *de novo* synthesis of 2′-deoxythymidine-5′-monophosphate (dTMP) [4].

Preclinical and clinical data suggest a link between the activation of cellular proliferative signalling pathways and resistance to chemotherapy [5]. Inhibition of the epidermal growth factor receptor (EGFR) has been shown in clinical settings to restore sensitivity to cytotoxic chemotherapy [6]. This suggests that the EGFR pathway contributes to chemoresistance [7]. Constitutive activating mutations in the *Ras* or *Raf* genes occur in approximately 45 and 10% of CRC, respectively [8, 9]. Clinical data indicates that patients with activating mutations in *Ras* or *Raf* have a worse prognosis [10]. In preclinical CRC models, activation of EGFR, IGFR or their downstream signalling pathways involving Ras, Raf, or Akt leads to increased proliferation and resistance to therapy [11], [12]. Growth promoting signals through these pathways lead to activation of c-myc and cyclin D1 [11], [13]. This in turn results in phosphorylation of retinoblastoma (Rb), releasing E2F transcriptional factors [14]. E2F1 plays a central role in cell proliferation through controlling the transition of cells from the G1 to S phase [15]. E2F1 also transcribes genes related to DNA synthesis and repair that are involved in cancer cell growth and resistance [16], including excision repair genes (ERCC-1), known to confer resistance to platinum agents, and TS [17]. Kasahara et al. [17] observed that TS expression correlates closely with transcriptional factor E2F1 expression in 23 colon cancer patient samples. High levels of TS expression have been associated with resistance to 5-FU [18]. Therefore, activation of E2F1 may provide a common pathway that explains at a molecular level the relationship between proliferation and resistance to therapy.

Heat shock protein 90 (HSP90) is a chaperone protein that regulates the stability and trafficking of several client proteins involved in cell proliferation [19]. Ganetespib is a small molecule inhibitor of HSP90 [20], which has shown encouraging single agent activity and a promising safety profile in early clinical trials [21]. Based on preclinical and clinical data, we hypothesize that inhibiting HSP90 activity will result in degradation of its client proteins and disruption of proliferative signalling pathways leading to cell cycle arrest and downregulation of TS, thereby sensitizing CRC cells to the effects of standard chemotherapy agents. To test this hypothesis, we evaluated the effect of inhibiting HSP90 by ganetespib or by genetic knockdown on proliferation and resistance to chemotherapy in CRC models.

## RESULTS

### Ganetespib induces G0/G1 cell cycle arrest and p21 and inhibits Cdk4, cyclin D1, and pRb in human colorectal cancer

**FIGURE 1:**
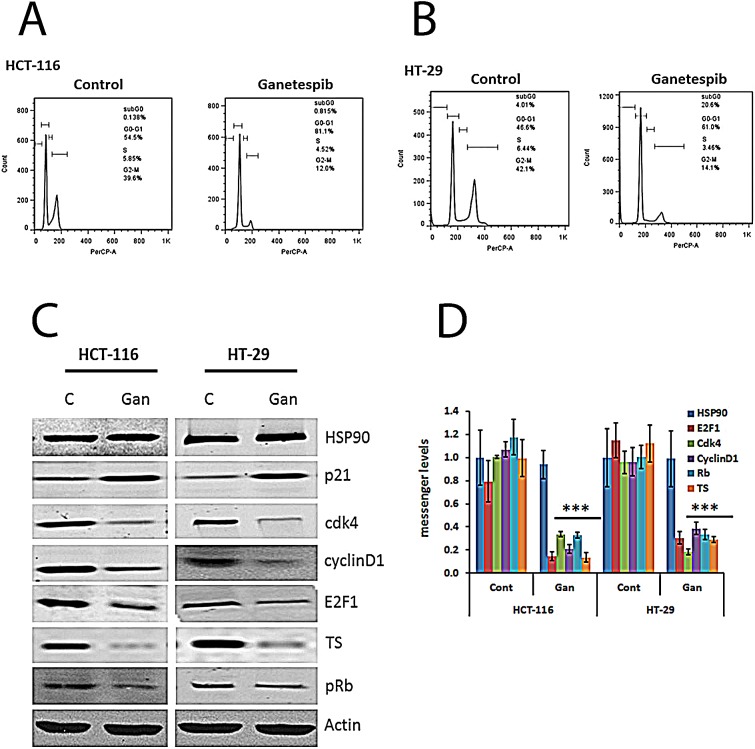
Cell cycle effects of ganetespib in colorectal cancer cell lines. Inhibition of HSP90 decreases levels of Cdk4, cyclin D1, pRb, E2F1, and TS and increases p21 (A & B) Cells were treated with vehicle (DMSO) or ganetespib (50nM) for 24 hours. Cell cycle arrest and the DNA content of CRC cells were measured by FACS analysis. Representative images from (a) HCT-116 and (B) HT-29 untreated and treated cells. (C) Cells were treated with vehicle (DMSO) or ganetespib (50nM) for 24 hours. Protein was extracted as described in the Methods section. Western blot analysis revealed increased expression of p21 and decreased expression of Cdk4, cyclin D1, pRb, E2F1, and TS in both cell lines treated with ganetespib. (D) Cells were treated with vehicle (DMSO) or ganetespib (50nM) for 24 hours. mRNA was extracted as described in the Methods section. mRNA was analyzed by qRT-PCR using primers for the indicated genes. Comparable qRT-PCR results were normalized with actin. Each value represents the mean ± standard deviation, obtained from determinations made on five cultures per experimental condition. qRT-PCR, quantitative real time-polymerase chain reaction. Ganetespib-treated HCT-116 and HT-29 cells showed significantly (*** p<0.001) decreased gene expression levels of Cdk4, cyclin D1, pRb, E2F1, and TS and significantly increased levels of p21 compared to controls.

Analysis of DNA content using flow cytometry revealed that ganetespib induced G0/G1 arrest in both cell lines (Fig. 1A & B). To understand the mechanism underlying this cell cycle effect, the protein and mRNA levels of cell cycle related HSP90 client proteins were evaluated. The protein and mRNA expression levels of CDK4 and cyclin D1 were significantly decreased in ganetespib treated CRC cells compared to controls in both cell lines (Fig. 1C & D). Since cyclin D1 and CDK4 are regulated by c-myc [22] and tumor suppressor gene *p21* [23], the expression of c-myc and p21 was measured in untreated and ganetespib treated CRC cell lines. The protein and mRNA expression levels of p21 were increased while the level of c-myc was decreased after ganetespib treatment (Fig. 1C & 1D). Cyclin D1 and CDK4 phosphorylate Rb [24], and as expected the downregulation of these molecules was associated with a decrease in the expression of pRb (Fig.1C). E2F transcription factors are key regulators of the cell cycle effects of Rb [25]. In both cell lines, treatment with ganetespib resulted in downregulation of E2F1 transcription factor (Fig. 1C & D). These molecular effects explain the G0/G1 arrest. One of the proteins transcribed by E2F1 is thymidylate synthase (TS) [17]. Treatment with ganetespib resulted in downregulation of the expression of TS at the protein and mRNA levels in both cell lines (Fig. 1C & 1D).

### Ganetespib inhibits EGFR and IGFR pathways in CRC cells

**FIGURE 2:**
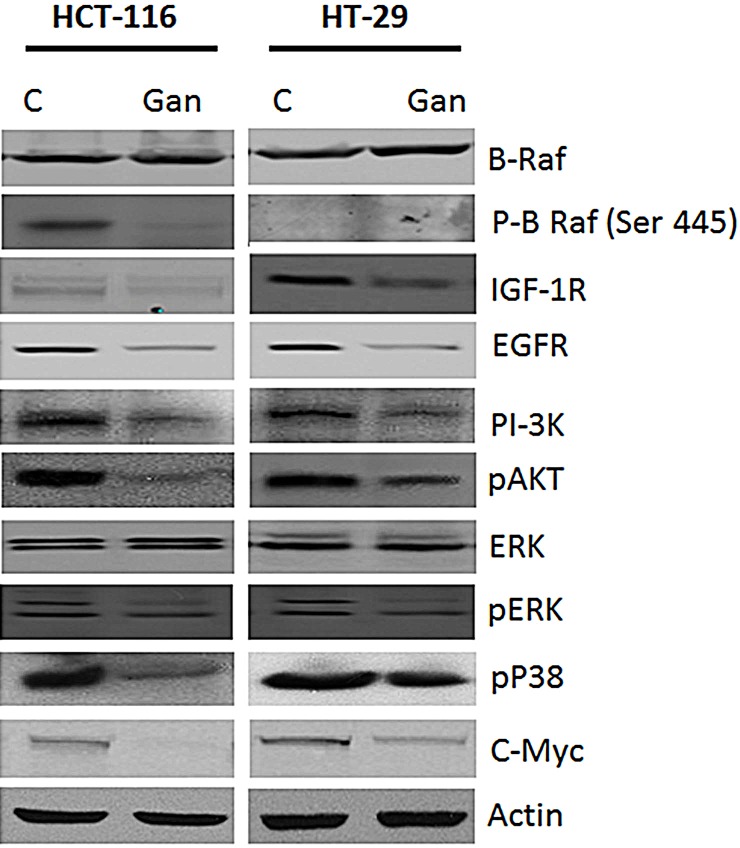
Effects of ganetespib on survival pathways in colorectal cancer cell line Treatment with ganetespib (50nM) decreased expression of IGF-1R, EGFR, PI-3K, p-Akt, ERK, p-ERK, p-P38, c-myc and p-BRAF as compared to vehicle (DMSO) in both HCT-116 and HT-29 cells.

Growth stimulatory signals are known to activate c-myc and cyclinD/ cdk leading to cell proliferation. HSP90 is a known chaperone protein for several proteins in the EGFR and IGFR signalling pathways. Treatment with ganetespib resulted in downregulation of EGFR and IGFR protein expression in both cell lines (Fig. 2). In addition, inhibition of HSP90 downregulated several key kinases in these signalling pathways including PI-3K, pAKT, pBRaf, JNK, pJNK, pP38, and pERK (Fig. 2) in both cell lines.

### Effects of HSP90 knock-down on survival and cell cycle regulatory molecules in human CRC cells

**FIGURE 3:**
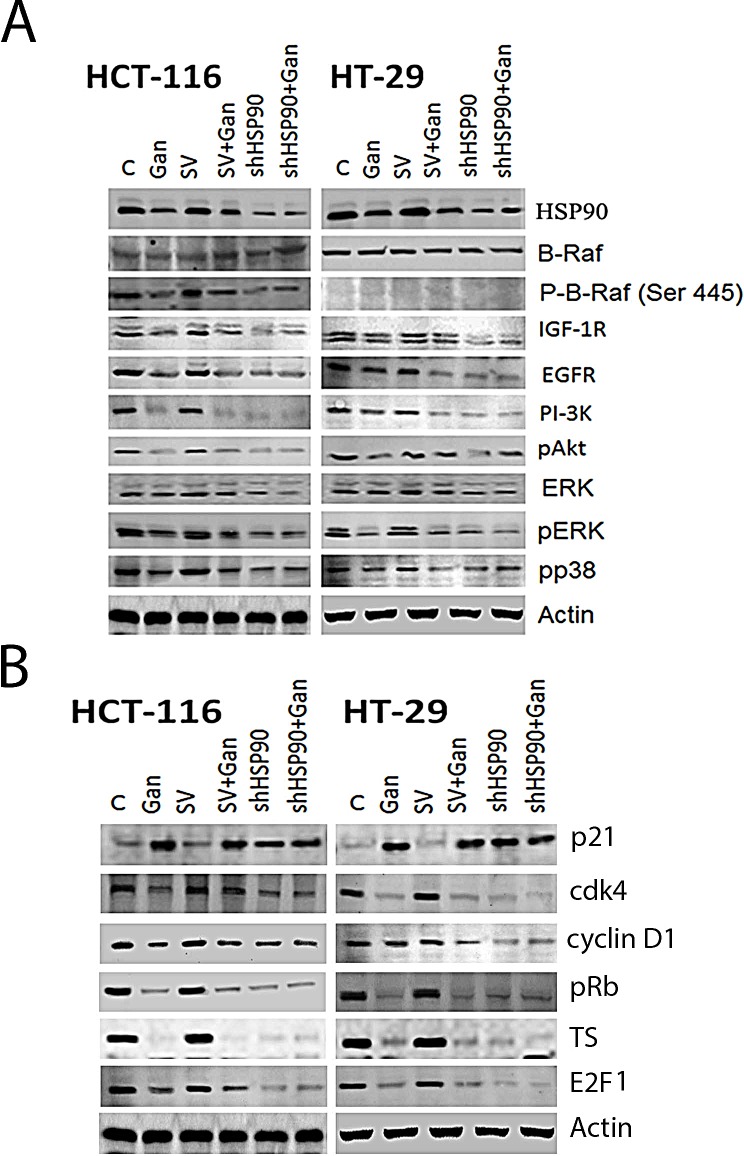
Effect of HSP90 knock-down on survival and cell cycle pathway regulatory molecules in human CRC cells Transfection of HCT-116 and HT-29 cells with HSP90 shRNA was performed as described in the methods section. Vehicle (DMSO) treatment and scrambled vector (SV) transfection were used as controls. Cells were treated with ganetespib 50nM for 24 hours. Western blot was performed as described in the Methods section. (A) Western blot of survival pathway molecules in HSP90 knock-down cells. Transfection with HSP90 shRNA resulted in a decrease in EGFR, PI-3K, pAKT, pMEK, pERK and c-Myc expression in both cell lines as compared to SV transfected cells. Treatment with ganetespib downregulated EGFR, PI-3K, pAKT, pMEK, pERK and c-Myc expression in both cell lines that were un-transfected or transfected with SV. Treatment with ganetespib did not affect the expression levels of PI-3K, pAKT, pMEK, pERK, and c-Myc in HSP90 shRNA transfected cells. No significant difference was observed between control and SV cells. (B) Western blot of cell cycle regulatory molecules in HSP90 knock-down cells. Transfection with HSP90 shRNA resulted in a decrease in Cdk4, cyclin D1, pRb, E2F1, and TS expression and upregulated p21 in both cell lines as compared to SV transfected cells. Treatment with ganetespib downregulated Cdk4, cyclin D1, pRb, E2F1, and TS expression and upregulated p21 in both cell lines that were un-transfected or transfected with SV. Treatment with ganetespib did not affect the expression levels of Cdk4, cyclin D1, pRb, E2F1, and TS in HSP90 shRNA transfected cells. No significant difference was observed between control and SV cells.

In order to confirm that the observed effects of ganetespib are mediated through HSP90, we transfected HCT-116 and HT-29 cells with HSP90 shRNA or scrambled vector. Transfection with HSP90 shRNA resulted in a decrease in HSP90 expression levels compared to the scrambled vector transfection and the non-transfected cell lines (Fig. 3A). The knockdown of HSP90 decreased the expression of EGFR, IGFR, PI-3K, pAKT, pP38, and pERK (Fig. 3A). Treatment with ganetespib did not affect the expression levels of EGFR, IGFR, PI-3K, pAKT, pP38, and pERK in HSP90 shRNA transfected cells (Fig. 3A). Similarly, transfection with HSP90 shRNA resulted in an increase in p21 expression levels compared to the scrambled vector transfection and the non-transfected cell lines (Fig. 3B). The knockdown of HSP90 decreased the expression of CDK4, cyclinD1, pRb, E2F1, and TS (Fig. 3B). Treatment with ganetespib did not affect the expression levels of CDK4, cyclinD1, pRb, E2F1, and TS in HSP90 shRNA transfected cells (Fig. 3B).

### Ganetespib inhibited survival and cell cycle regulatory molecules in human CRC xenografts

**FIGURE 4:**
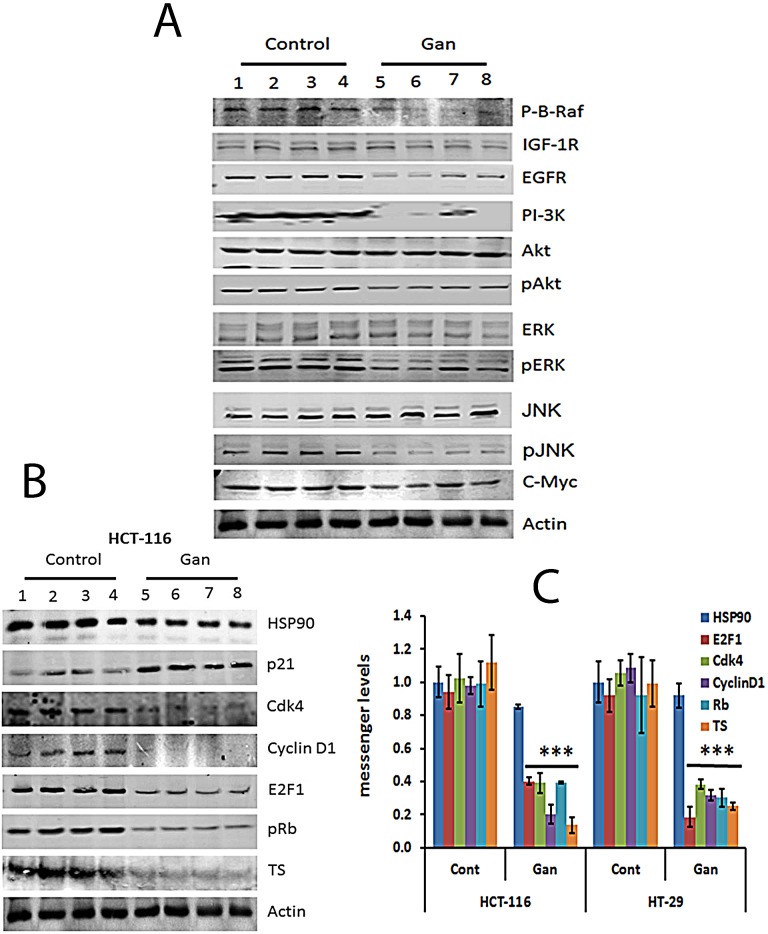
Treatment with ganetespib inhibits regulatory molecules involved in survival and cell cycle molecular pathways in HCT-116 and HT-29 xenografts (A) Extracts from resected tumors from 4 vehicle or 4 ganetespib-treated animals were analyzed by Western blot as detailed in methods. Decreased protein expression of EGFR, PI-3K, pAKT, pMEK, pERK and c-myc was observed in tumors treated with ganetespib. (B) Extracts from resected tumors from 4 untreated or 4 ganetespib-treated animals, were analyzed by Western blot as detailed in methods. Decreased protein expression of Cdk4, cyclin D1, pRb, E2F1, and TS and increased levels of p21 were observed in tumors treated with ganetespib. (C) Extracts from resected tumors from 4 untreated or 4 ganetespib treated animals, were analyzed by qRT-PCRas detailed in methods. Comparable qRT-PCRresults were normalized with actin. Significant (*p*<0.001) decrease in the mRNA expression levels of Cdk4, cyclin D1, pRb, E2F1, and TS were observed in tumors treated with ganetespib as compared to control. An increased mRNA expression level of p21 was observed in treated tumors as compared to control. Each value represents the mean ± standard deviation, *** p<0.001.

Tumors resected from animals treated with ganetespib or controls were then examined for protein and mRNA expression. Similar to the *in vitro* effects, ganetespib significantly downregulated proliferative signalling pathways with decreased protein expression of EGFR, IGFR, pAkt, PI3K, pERK, Raf and pJNK (Fig. 4A). Treatment with ganetespib also downregulated the expression of cell cycle related proteins and messages including cyclinD1, cdk, pRb, and E2F1 and increased the expression of p21 (Fig. 4B & C). Treatment with ganetespib also downregulated the expression of TS in both xenograft models (Fig. 4B & C). These results corroborate our *in vitro* findings that HSP90 inhibition alters growth signalling, leading to cell cycle arrest and downregulation of S-phase related proteins such as TS (Fig. 1).

### Ganetespib sensitizes human CRC cell lines and tumor xenografts to 5FU and oxaliplatin

**FIGURE 5:**
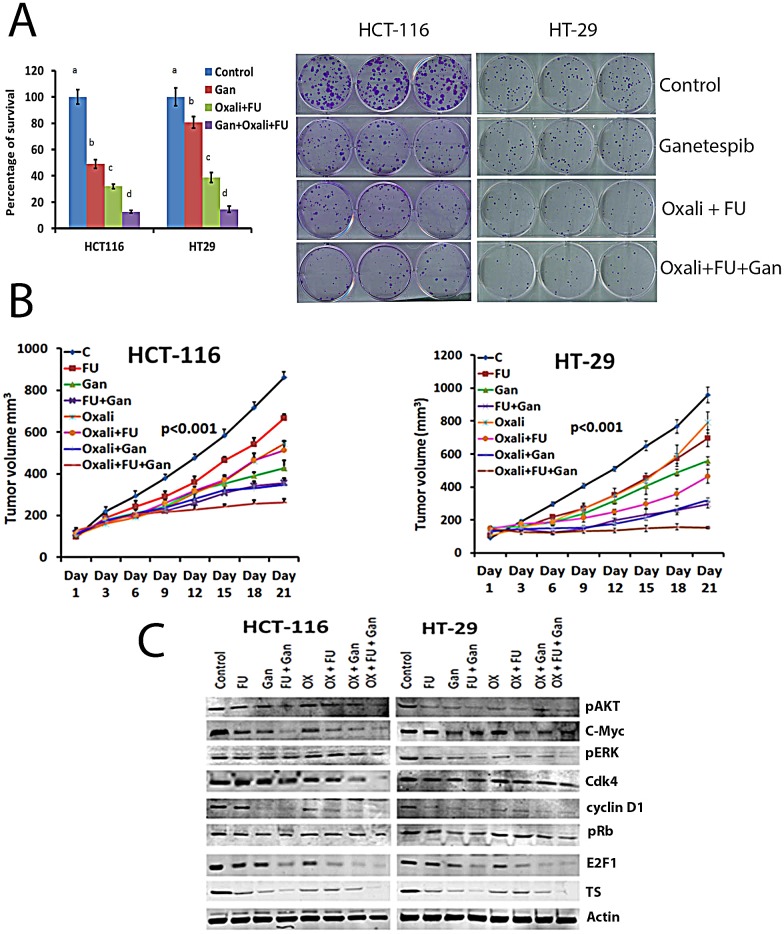
Ganetespib sensitizes CRC cell lines and tumors to oxaliplatin and 5-FU (A) Colony formation assay was performed. Cells were treated with vehicle, 5FU/oxaliplatin (10/2.5 μM), ganetespib (50nM) or the combination as described in the Materials and Methods section. A significant (*p*<0.001) decrease in colony formation was observed in oxaliplatin/5FU (c) treated cell lines as compared to control (a). A significant (*p*<0.001) decrease in colony formation was observed in cells treated with ganetespib, oxaliplatin and 5FU (d) as compared to cells treated with oxaliplatin and 5FU (c). Bars with different letters = *p*<0.001; ANOVA. (B) Treatment with ganetespib sensitizes CRC xenografts to chemotherapy and decreases tumor growth as well as inhibits molecular pathways involved in survival and cell cycle. Nude mice had HCT-116 or HT-29 cells implanted subcutaneously as described in the Materials and Methods section. Tumor volume was measured once every three days. Tumors were resected from animals at the completion of treatment. No significant difference was observed in body weight between control and treated animals. A significant (p<0.001) decrease in the average tumor volume was observed in animals treated with ganetespib as compared to control. A significant decrease was observed in tumors from animals treated with ganetespib plus 5FU/oxaliplatin as compared to oxaliplatin and 5FU. (C) Extracts from resected tumors from untreated or treated animal tumors, were analyzed by Western blot as detailed in methods. Decreased protein expression of pAKT, pERK and c-Myc, Cdk4, cyclin D1, pRb, E2F1 and TS was observed in tumors treated with ganetespib combination with 5-FU and oxaliplatin compared to individual treatment and/or controls.

A clonogenic assay was used to evaluate the effect of combining ganetespib with the standard chemotherapy agents 5FU and oxaliplatin. In both cell lines, 5FU and oxaliplatin resulted in significant inhibition of colony formation when compared to untreated cells (Fig. 5A). The addition of ganetespib to 5FU and oxaliplatin resulted in a significantly greater reduction in colony formation when compared to 5FU and oxaliplatin treated cell lines (Fig. 5A). This potentiation was observed in both cell lines.

Both 5-FU and oxaliplatin inhibited tumor growth as compared to the control group in HT-29 and HCT-116 xenografts (Fig. 5B). The combination of 5FU and oxaliplatin was significantly (p<0.001) more effective than either agent alone in both models. The addition of ganetespib to 5FU, oxaliplatin or the combination resulted in a significant (p<0.001) increase in tumor growth inhibition as compared to the tumors treated with chemotherapy alone. These effects were observed in both xenografts.

Finally, we evaluated the effects of treatment with 5FU and oxaliplatin alone, combined, and in combination with ganetespib on the expression of pERK, c-myc, cyclin D1, pRb, E2F1 and TS (Fig. 5C). Combining ganetespib with 5FU, oxaliplatin or the combination resulted in significant (p<0.001) downregulation of proliferative and cell cycle signalling pathways including inhibition of pERK, p21, cdk4, c-myc, cyclin D1 and E2F1 (Fig. 5C). This was associated with downregulation of expression of TS in the ganetespib treated tumors (Fig. 5C).

## DISCUSSION

Sustaining proliferative signalling is a hallmark of cancer [26]. Proliferative signalling pathways are commonly dysregulated in colorectal cancer. For example, the EGFR is known to be overexpressed and activated in CRC tumors and cells [27], and its inhibition results in restoration of sensitivity to chemotherapeutic agents [6]. Similarly, IGF-1 and IGF-1R are known to be overexpressed in CRC [6]. Chemoresistant colorectal cell lines have been shown to have an increased expression and activation of IGF-1R [28]. Furthermore, inhibition of IGF-1R has been shown to sensitize CRC cell lines to the effects of chemotherapy. Proliferative signalling pathways downstream of the EGFR and IGF-1R are also commonly dysregulated in CRC [10]. Activating mutations in *Ras* and *Raf* genes are known to occur in approximately 45% and 15% of CRC, respectively [29], [30]. These mutations have been associated with poor overall survival as well as resistance to chemotherapy [31], [32]. Activation of these proliferative signalling pathways results in activation of ERK leading to phosphorylation and activation of cyclin D1[27], [33]. Mutations in the adenomatous polyposis coli (APC) gene are known to occur in approximately 60% of all CRC patients [32] and result in activation of β-catenin/Tcf4 which in turn activates cyclin D1 [30]. Cyclin D1 together with its binding partners, cyclin-dependent kinase (CDK)-4 and CDK6, forms active complexes that promote G1- to S-phase progression by phosphorylating and inactivating the Rb protein [29]. E2F1 transcription factor is released from pRb allowing it to translocate to the nucleus and transcribe genes related to DNA replication, DNA repair and cell cycle regulation [34]. Therefore, the cyclin D1 and E2F1 pathways represent a rational target for therapeutic intervention because their activation is common in CRC patients, induces proliferation, and may contribute to resistance to therapy.

**FIGURE 6:**
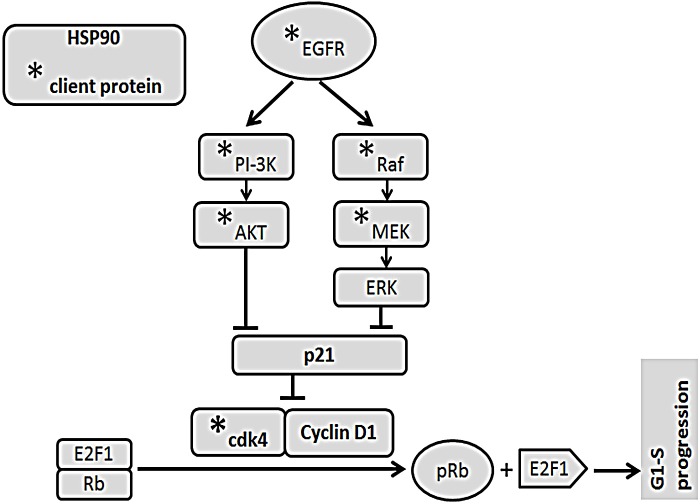
Signalling mechanism EGFR activates PI-3K/Akt and Ras/Raf/ERK signalling pathways and stabilized by HSP90. EGFR, PI3K, AKT, MEK, cdk4 are client proteins of HSP90. HSP90 induces cell cycle progression through modulation of EGFR/PI-3K/Akt and Raf/ERK pathways as well as stabilization of cdk4/Cyclin D1. Functional inhibition of HSP90 by ganetespib suppresses cell cycle progression by inhibiting the survival pathways and phosphorylation of Rb.

Given the complexity of the proliferative signalling pathways, targeting these pathways would require the inhibition of multiple key proteins. In this study, we have demonstrated that inhibiting HSP90 with ganetespib or by genetic knockdown results in inhibition of proliferative pathways at multiple levels including surface growth factor receptors (EGFR, IGF-1R) and downstream pathways (PI3K, pAkt, Raf, p38, pJNK, and pERK; Fig. 6). These effects were seen in two CRC cell lines, one with *Ras* mutation (HCT-116) and one with a *B-Raf* mutation (HT-29). The concentrations of ganetespib used in these experiments were based on the previously identified IC_50_ for HCT-116 and HT-29 cell lines [35]. This concentration is comparable to the concentration achieved in phase I clinical trials [36]. The effects of ganetespib on proliferative pathways were also seen *in vivo* using tumor xenograft models.

The inhibition of HSP90 resulted in decreased phosphorylation of Rb and consequently decreased release of E2F1 and arrest of cell cycle at G0-G1 (Fig. 6). Among the proteins transcribed by E2F1, we focused our attention on TS, the target for 5FU. TS has been shown to be a prognostic factor for patients with stage II and III disease [37]. Clinical studies have shown an association between TS expression and resistance to 5FU in advanced stage CRC [18, 38]. Similar relationships between TS expression and resistance to 5FU have been observed in pancreatic [39], and head and neck[40] cancers. In addition to expression, structural alterations in TS that affect its affinity to 5FU may contribute to resistance. Such a resistant variant of TS has been shown to occur in the HCT-116 cell line [41]. In this study, we demonstrate that inhibition of HSP90 can downregulate the transcription and expression of TS in HT-29 and HCT-116 cell lines. Modulation of TS represents a novel mechanism that may further explain the observed sensitization of CRC[42] and bladder cancer cell lines[43] to HSP90 inhibitors. Treatment with 5FU has been associated with induction of TS expression leading to acquired resistance to 5FU [4]. In the current study, we did not observe any increase in TS expression in tumors from animals treated with 5FU and ganetespib, suggesting that inhibition of HSP90 may delay or prevent this mechanism of acquired resistance.

In addition to the effects on proliferation and TS expression, ganetespib has been shown to be a potent antiangiogenic agent in CRC models [44]. The antiangiogenic effects are mediated through inhibition of HIF-1α and STAT-3, leading to downregulation of vascular endothelial growth factor (VEGF) transcription [44]. The effects of ganetespib on VEGF expression were also demonstrated using pre and post treatment biopsies from patients with rectal cancer [44]. Randomized clinical trials have already demonstrated the benefit of combining VEGF targeting agents with chemotherapy in stage IV CRC.[45, 46] Ganetespib also inhibits epithelial to mesenchymal transition (EMT) in CRC cell lines [35]. EMT is known to be associated with increased invasiveness and resistance to therapy [35]. Based on its anti-invasive, antiangiogenic and anti-proliferative effects, we evaluated the combination of ganetespib with standard chemotherapy in cell line and animal models. The results confirm that ganetespib can potentiate the growth inhibition of 5FU and oxaliplatin in both *Ras* and *Raf* mutated cell lines. Our results are in agreement with previously published literature. Moser et al. [47] had demonstrated that 17-DMAG, an HSP90 inhibitor, can sensitize p53 deficient CRC cells to the effects of oxaliplatin.

In conclusion, we have demonstrated that inhibition of HSP90 can sensitize CRC cells to the effects of chemotherapy through inhibition of proliferative signalling pathways and downregulation of TS. Ganetespib has already demonstrated an excellent safety profile in clinical trials. In a phase II trial of ganetespib in heavily pre-treated patients with stage IV CRC, 2/15 patients had stable disease and no objective responses were seen [48]. Although single agent ganetespib may have limited activity in CRC, combining it with 5FU and oxaliplatin is a rational approach based on the safety profile, mechanism of action and the preclinical data from animal models. Elevated baseline TS expression may be a useful clinical biomarker to select patients who might benefit from combining ganetespib with fluoropyrimidine.

## MATERIALS AND METHODS

### Cells and reagents

CRC cell lines HCT-116 and HT-29 were obtained from American Type Culture Collection (ATCC, Manassas, VA) in 2011. Characterization and authentication were reported by ATCC in the accompanying certificate of analysis. Cultures had been validated by ATCC to be Mycoplasma-free, expressed only basal epithelial cell markers and unique human DNA profile. They were subsequently cultured in McCoy’s 5A medium contain 1.5 mM L-glutamine and 2200 mg/L sodium bicarbonate (Cat # ATCC^®^ 30-2007^TM^, ATCC, Manassas, VA) supplemented with 10% fetal bovine serum (Cat # 4135; Sigma-Aldrich, St. Louis, MO), 50 units/ml penicillin, and 50 μg/ml streptomycin (Life Technologies, Inc., Frederick, MD). Cells were incubated at 37°C in a humidified 5% CO_2_ atmosphere. In our previous experiments, we demonstrated that the IC_50_ of ganetespib in both HT-29 and HCT-116 cells was 50 nM.[35] Specific antibodies against HSP90, PI-3K, Akt, pAkt, EGFR, pERK, MEK, cMyc, CDK4, cyclinD1, p21, pRb, E2F1, TS, β-actin, and HRP (horseradish peroxidase) conjugated secondary antibodies were purchased from Cell Signalling (Beverly, MA) and Santa Cruz Biotechnology (Dallas, TX). HSP90 shRNA and scrambled vector (SV) were obtained from Santa Cruz Biotechnology (Dallas, TX). Ganetespib was obtained from Synta Pharmaceuticals (Lexington, MA). 5-Fluorouracil (5FU) and oxaliplatin were purchased from Sigma Aldrich (Saint Louis, MO).

### Clonogenicity assay

For this experiment, HCT-116 and HT-29 cells were treated with vehicle, ganetespib (50nM), 5FU (10 μM ) /oxaliplatin (2.5 μM) or ganetespib (50nM)/ 5FU (10 μM) /oxaliplatin (2.5 μM). HCT-116 and HT-29 cells were seeded (100 cells/well) in triplicate in 6-well plates. The media was changed 24 hours after platting and the cells were treated with either vehicle (DMSO) or ganetespib. Twenty four hours after first treatment, media was changed and the cells were either treated with oxaliplatin and 5FU or vehicle for another 24hr. Media was changed once every 3 days without treatment. On the 12th day, the medium was removed and cell colonies were stained with crystal violet (0.1% in 20% methanol). Colony numbers were assessed visually and colonies containing >50 normal-appearing cells were counted. Assays were performed in triplicate.

### Flow cytometry

Cells were treated with vehicle (DMSO) or ganetespib (50nM) as described above and were harvested after 24 hours and fixed in 70% ethanol for 1 hour at 4°C. The cells were stained with propidium iodide (BioSure, Grass Valley, CA) for 15 min in the dark and FACS analysis was performed using a Becton-Dickinson FACS Calibur flow cytometer (BD Biosciences, Heidelberg, Germany) per the manufacturer’s instructions. Assays were performed in duplicate.

Results were analyzed with Flow-Jo (TreeStar Inc., Ashland, OR) software.

### Western blotting and immunoprecipitation

Western blotting was performed as previously described [35]. Briefly, CRC cells were treated with vehicle (DMSO) or ganetespib (50 nM) for 24 hours, then harvested and lysed in RIPA protein extraction buffer containing protease inhibitors (Sigma-Aldrich, Saint Louis, MO). Protein extracts were then analyzed by Western blotting. Briefly, equal amounts of protein fractions of lysates were resolved over SDS-PAGE and transferred onto PVDF membrane. Membranes were incubated with primary antibodies followed by HRP-conjugated secondary antibodies. Bound antibodies were visualized using enhanced chemiluminescence (ECL System; Amersham, Arlington Heights, IL). To confirm equal loading, membranes were verified by re-probing with an antibody specific for the housekeeping gene, anti-*β*-actin.

### Quantitative real time-polymerase chain reaction (qRT-PCR)

Total RNA was isolated using TRIzol reagent (Invitrogen Corporation, Carlsbad, CA). The reverse transcription (RT) step was performed with MultiScribe^TM^ reverse transcriptase (Applied Biosystems, USA). To determine the transcript levels from cDNA, qRT-PCR was carried out with 1 μL of cDNA using specific primers (gene and primer details given in Table 1). The qRT-PCR conditions were as follows: initial denaturation at 95 ^0^C for 3 min, followed by 30 cycles at 95 ^0^C for 1 min, 57 ^0^C for 30 s (depending on primer sets), 72 ^0^C for 1 min, followed by a final extension at 72 ^0^C for 7 min. Melting curve analysis verified a single product. Relative quantities were calculated and standardized by comparison to actin [35].

**Table 1:** Gene specific primers for qRT-PCR analysis

Gene Accession #	Name	Sequence	Size (bp)
NM_017963	Hsp90	5′ TTC AGA CAG AGC CAA GGT GC 3′	167
		5′ CAATGA CAT CAA CTG GGC AAT 3′	
NM_000075	CDK4	5′- GAG TGT GAG AGT CCC CAA TG -3′	181
		5′- ATG CTC AAA CAC CAG GGT TA -3′	
NM_053056	CyclinD1	5′- TTC AAA TGT GTG CAG AAG GA -3′	221
		5′- GGG ATG GTC TCC TTC ATC TT -3′	
NM_005225	E2F1	5′- AGT TCA TCA GCC TTT CCC CA -3′	417
		5′- TTC ACC TTC ATT CCC CGG TA -3′	
NM_000321	Rb1	5′- AAA GGA CCG AGA AGG ACC AA -3′	296
		5′- CTG GGT GCT CAG ACA GAA GG -3′	
NM_001071	TS	5′- CTGCCAGCTGTACCAGAGAT-3′	142
		5′- ATGTGCATCTCCCAAAGTGT-3′	

### Transfection studies

Transfection studies were performed as previously described [35]. For HSP90 knockdown studies, HCT-116 and HT-29 cells were plated at sub-confluent densities. The following day, the HCT-116 and HT-29 cells were transfected with either SV or HSP90 shRNA by using Lipopectamine 2000 transfection reagent (Invitrogen). Cells were harvested at 48 h for Western blot and qRT-PCR analyses. Knockdown of HSP90 was confirmed by Western blot and qRT- PCR analyses.

### *In vivo* tumor growth inhibition study

Tumor growth inhibition study was performed as previously described [35]. Five to six-week-old female nude mice were obtained (Harlan, Indianapolis, Indiana) and maintained in a pathogen-free environment. All *in vivo* procedures were approved by Emory University’s Institutional Animal Care and Use Committee. HCT-116 and HT-29 cells (2 × 10^6^) were subcutaneously grafted into the mice and were divided into 8 groups with 5 mice in each group. When tumors reached 100 ± 10 mm^3^, groups (3, 4, and 7) received ganetespib (100 mg/kg body weight) intravenously once weekly for three weeks. Groups (2, 4, 6, and 8) received 5FU (30mg/kg) and groups (5, 6, 7, and 8) received oxaliplatin (5mg/kg) intravenously once weekly (after 24hr of ganetespib treatment) for three weeks. None of the animals died from the treatment. Every third day, tumor size was measured using vernier caliper scale for a total of three weeks, when the animals were anesthetized with ketamine and sacrificed by cervical dislocation. Tumor growth inhibition was determined as previously described [35]. Tumor samples were used for Western blot and qRT-PCR analysis.

### Statistical analysis

Clonogenicity and tumor growth data were analyzed using one-way ANOVA with by Neumann-Keuls method of Instat. Data are shown in the form of mean ± SD, which was obtained from five individual repetitions. qRT-PCR data were analyzed using unpaired Student t test in for comparison between control and ganetespib treated cells. Data are showed in the form of mean ± SD, which was obtained from five individual repetitions. All statistical analyses were completed using GraphPad Prism software (La Jolla, CA, USA).

### Authors’ Contribution Preclinical experiments

Nagaraju GP, Olatunji B. Alese. Experimental design: Nagaraju GP, R Diaz, B El-Rayes. Manuscript preparation: Nagaraju GP, B El-Rayes. Manuscript review and editing: All.

### Disclosure of Potential Conflict of Interest

B. El-Rayes and R. Diaz receive research support from Synta Pharmaceuticals. All other co-authors have no conflict to declare.

### Financial support

This work was supported by Georgia Cancer Coalition (#00026700), Kennedy Award (#00015855). Cancer.gov number: NCT 01554969
